# Roles of the C-Terminal Amino Acids of Non-Hexameric Helicases: Insights from *Escherichia coli* UvrD

**DOI:** 10.3390/ijms22031018

**Published:** 2021-01-20

**Authors:** Hiroaki Yokota

**Affiliations:** Biophotonics Laboratory, The Graduate School for the Creation of New Photonics Industries, 1955-1, Kurematsu-cho, Nishi-ku, Hamamatsu, Shizuoka 431-1202, Japan; yokota@gpi.ac.jp

**Keywords:** protein–nucleic acid interactions, helicase, single-molecule fluorescence imaging, C-terminal amino acids, protein assemblies, enzyme function

## Abstract

Helicases are nucleic acid-unwinding enzymes that are involved in the maintenance of genome integrity. Several parts of the amino acid sequences of helicases are very similar, and these quite well-conserved amino acid sequences are termed “helicase motifs”. Previous studies by X-ray crystallography and single-molecule measurements have suggested a common underlying mechanism for their function. These studies indicate the role of the helicase motifs in unwinding nucleic acids. In contrast, the sequence and length of the C-terminal amino acids of helicases are highly variable. In this paper, I review past and recent studies that proposed helicase mechanisms and studies that investigated the roles of the C-terminal amino acids on helicase and dimerization activities, primarily on the non-hexermeric *Escherichia coli* (*E. coli*) UvrD helicase. Then, I center on my recent study of single-molecule direct visualization of a UvrD mutant lacking the C-terminal 40 amino acids (UvrDΔ40C) used in studies proposing the monomer helicase model. The study demonstrated that multiple UvrDΔ40C molecules jointly participated in DNA unwinding, presumably by forming an oligomer. Thus, the single-molecule observation addressed how the C-terminal amino acids affect the number of helicases bound to DNA, oligomerization, and unwinding activity, which can be applied to other helicases.

## 1. Introduction

Helicases are enzymes that unwind nucleic acids using energy derived from NTP hydrolysis and fulfill essential functions in the maintenance of genome integrity, including DNA replication, repair, and recombination. Several parts of the amino acid sequences of helicases are very similar, and these quite well-conserved amino acid sequences are termed the “helicase motifs” [1]. These motifs allow us to classify helicases into six superfamilies (SFs). The number of conserved helicase motifs for SF1 and SF2 is at least seven, and they share common sequences [2]. These conserved motifs, which are essential to adenosine triphosphate (ATP) hydrolysis and nucleic acid binding and unwinding, are commonly placed not at N- and C-termini, but in the middle of the amino acid sequences. In contrast, the sequence and length of the N-terminal and C-terminal amino acids of helicases are highly variable [3], though these terminal regions are in charge of various protein functions.

The six SFs can be categorized by the number of molecules that are involved in helicase functions. SF3–6 helicases function through the formation of a hexameric ring around DNA [4,5], while SF1 and SF2 helicases function in a non-hexameric form.

Tertiary structures resolved by X-ray crystallography [6,7,8,9] indicate that SF1 helicases share four structural domains (1A, 1B, 2A, and 2B) and function using a common mechanism. However, two conflicting DNA-unwinding models, dimeric-helicase and monomeric-helicase models have been proposed for non-hexameric helicases.

The N-terminal and C-terminal amino acids of helicases, which are not conserved in SF1 and SF2, serve various protein functions. In particular, many studies demonstrated that the C-terminus of helicases plays essential roles in nucleic acid binding and dimerization activities.

In this paper, I review past and recent studies that proposed helicase mechanisms and studies that investigated the roles of the C-terminal amino acids of helicase and dimerization activities, primarily on the non-hexermeric *Escherichia coli* (*E. coli*) SF1 UvrD helicase. I center on my recent study of single-molecule direct visualization of a UvrD mutant lacking the C-terminal 40 amino acids (UvrDΔ40C) [10] used in studies proposing the monomer helicase model. The study demonstrates that two or three UvrDΔ40C molecules jointly participate in DNA unwinding, presumably by forming an oligomer, similar to that of wild-type UvrD. Thus, the results should settle the controversy of the monomer/oligomer helicase models, address the role of C-terminal amino acids in binding and unwinding DNA, and elucidate their effects on helicase and dimerization activities.

## 2. Helicase Superfamily

The sequence similarity of the helicase motifs among helicases allows us to classify helicases into six superfamilies (SF1–6). The six SFs can be categorized by the number of molecules that are involved in helicase function. SF3–6 helicases function through the formation of a hexameric ring around DNA [4,5], whereas SF1 and SF2 helicases function in a non-hexameric form.

Non-hexameric SF1 and SF2 helicases consist of at least seven conserved amino acid motifs, “helicase motifs” (I, Ia, II, III, IV, V, and VI) [1,11]. The seven conserved motifs are partially varied between SF1 and SF2. For example, the primary structures and locations of helicase motif III in SF1 and SF2 helicases are not homologous.

### 2.1. Superfamily 1 Helicases

**Figure 1:**
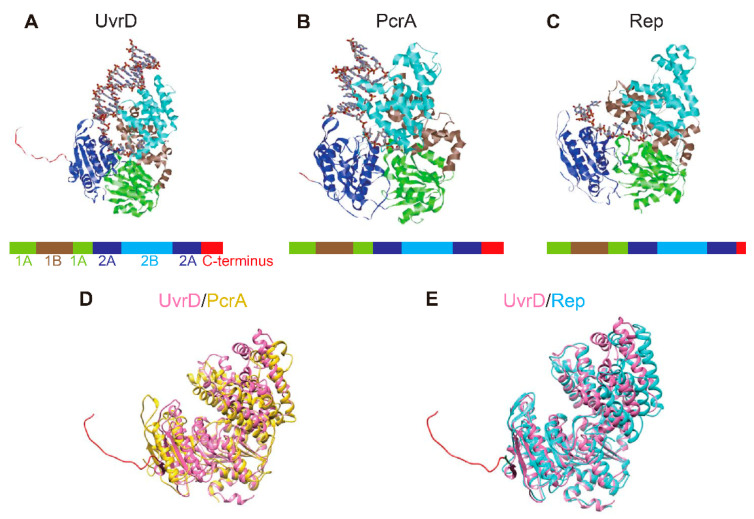
Structural similarity among SF1 helicases. (**A**–**C**) Crystal structures of *Escherichia coli* (*E. coli*) UvrD (PDB code 2IS4) (**A**), *Geobacillus stearothermophilus* (*G. stearothermophilus*) PcrA (PDB code 3PJR) (**B**), and *E. coli* Rep (PDB code 1UAA) (**C**), which were complexed with DNA. The 1A, 1B, 2A, and 2B domains and unstructured C-terminus are colored in blue, brown, cyan, green, and red, respectively. Each primary structure diagram is depicted below the tertiary structure. (**D**,**E**) Structural superposition of UvrD and PcrA (**D**), and UvrD and Rep (**E**). The unstructured C-termini of UvrD and PcrA are colored in red and blue, respectively. Molecular graphics and analyses performed with UCSF Chimera, developed by the Resource for Biocomputing, Visualization, and Informatics at the University of California, San Francisco, with support from NIH P41-GM103311 [13].

**Figure 2:**
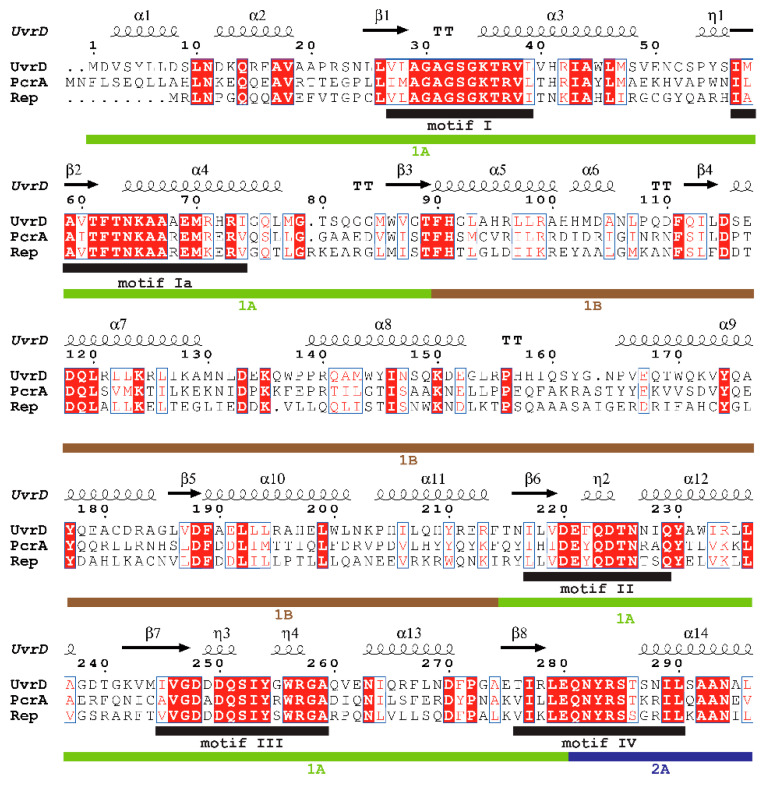

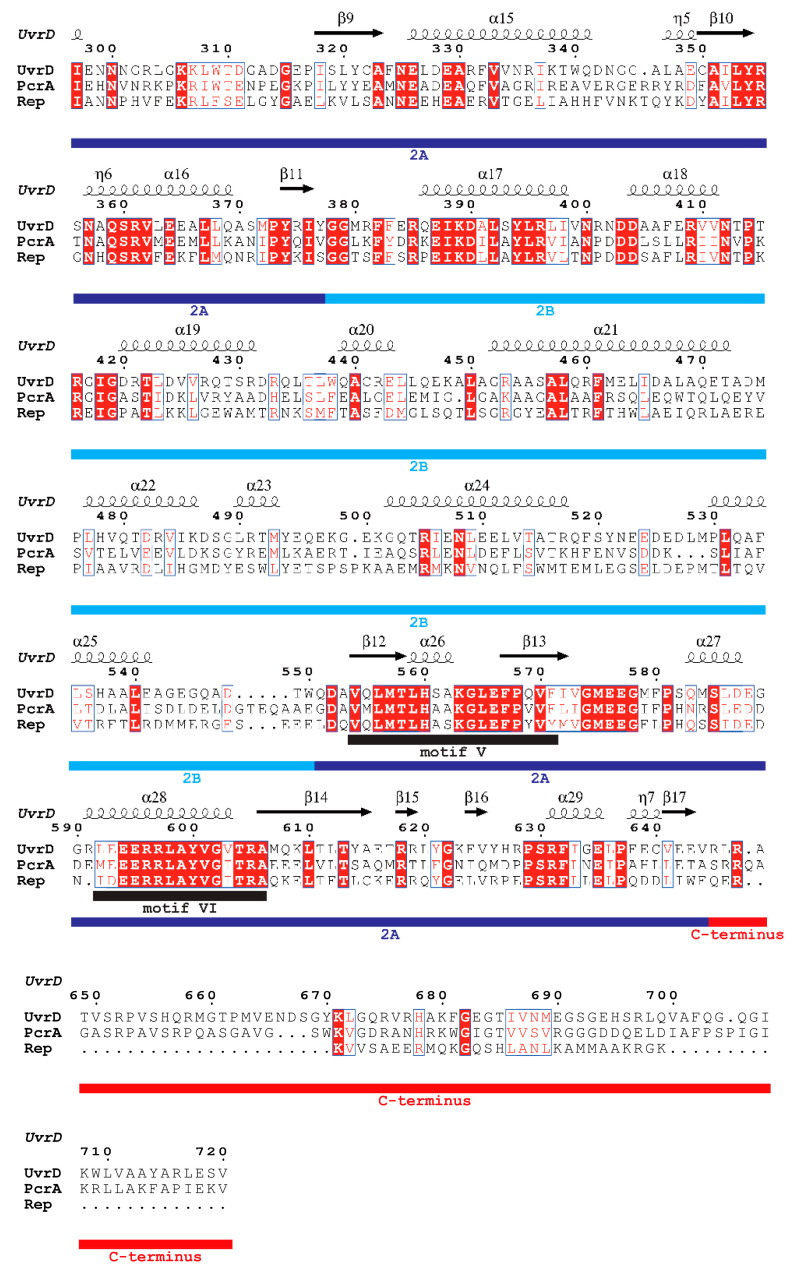
Secondary structure-based sequence alignment of SF1 helicases. The alignment and color representation were performed using ClustalW2.1 (https://www.genome.jp/tools-bin/clustalw) [14] and ESPript 3.0 web-based server (http://espript.ibcp.fr/ESPript/ESPript/) [15], respectively. All of the conserved residues are boxed with blue frames, and the fully conserved residues are colored white with a red background, whereas the less conserved residues are colored red. Helices and strands are labeled according to the UvrD structure. α-helices, 3_10_-helices, and π-helices are indicated as medium, small, and large squiggles, respectively. β-strands are displayed as arrows, strict β-turns as TT letters, and strict α-turns as TTT. The I, Ia, II, III, IV, V, and VI helicase motifs [6] as well as the 1A, 1B, 2A, and 2B domains and unstructured C-terminus of UvrD, are indicated.

Superfamily 1 is a major class of helicases that are essentially involved in nucleid acid metabolism [3,12]. Some of the well-studied SF1 helicases are *E. coli* UvrD and Rep, and *Bacillus stearothermophilus* (*B. stearothermophilus*) or *Geobacillus stearothermophilus* (*G. stearothermophilus*) PcrA, whose primary structures are highly homologous (approximately 40%), and these have been resolved by X-ray crystallography [6,7,8,9]. These structures revealed that these three SF1 helicases contain four structural domains (1A, 1B, 2A, and 2B). Their structures, resolved by X-ray crystallography, are shown in Figure 1A–C. The structural similarity is seen from the structural superposition of UvrD and PcrA (Rep) (Figure 1D,E). Figure 2 shows the results obtained by the secondary structure-based sequence alignment of UvrD, PcrA, and Rep. The I, Ia, II, III, IV, V, and VI helicase motifs [6] as well as the 1A, 1B, 2A, and 2B domains and unstructured C-terminus of UvrD are indicated.

### 2.2. Superfamily 2 Helicases

The SF2 is another main superfamily including various DNA helicases as well as several subfamilies of RNA helicases. Most RNA helicases are categorized as SF2 and are members of the Asp-Glu-Ala-Asp (DEAD)-box, the Asp-Glu-Ala-His (DEAH), and the DExH (where x can be any amino acid) subfamilies. The four-letter names of the subfamilies come from the sequence of helicase motif II.

### 2.3. Proposed Models for the Functional Unit of Some SF1 and SF2 Helicases

Although non-hexameric SF1 and SF2 helicases have common amino acid motifs, the opposing dimeric-helicase and monomeric-helicase models have been proposed for non-hexameric SF1 and SF2 helicases. On one hand, the dimeric-helicase model has been proposed for SF1 helicases (*E. coli* UvrD [10,16,17,18,19,20,21], Rep [8,22,23], and TraI [24] helicases, and *B. subtilis* PcrA [25] helicase) and SF2 helicases (*E. coli* RecQ [26], hepatitis C viral NS3 [27], and DEAD-box RNA helicases, including *E. coli* CsdA [28] and RhlB [29], *G. stearothermophilus* CshA [30], a and *Thermus thermophilus* (*T. thermophilus*) Hera [31]). On the other hand, the monomeric-helicase model was proposed for SF1 UvrD [6,32], PcrA [9,33], TraI [34], phage T4 Dda [35,36], and SF2 RecQ [37,38,39] and NS3 helicases [40]. Table 1 lists the cellular functions of some SF1 and SF2 helicases and the proposed models for their functional units.

The dimeric-helicase and monomeric-helicase models have been proposed mostly by structural and biochemical studies, including X-ray crystallography, ATPase assays, stopped-flow method-based single- and multiple-turnover DNA-unwinding assays, size exclusion chromatography, and analytical ultracentrifugation. The models for some helicases were tested by single-molecule measurements as this paper illustrates for SF1 UvrD helicase in the following sections. Note that the solution conditions, such as NaCl concentration, pH, glycerol concentration, and temperature, affect the self-assembly of helicases. For UvrD helicase, Maluf et al. extensively investigated the effects of such solution conditions on the self-assembly states. They quantitively characterized the self-assembly equilibria of wild-type UvrD as a function of NaCl and glycerol concentrations as well astemperature using analytical ultracentrifugation and concluded that a lower NaCl concentration, a lower pH, a lower glycerol concentration, and a higher temperature were favorable for UvrD oligomer formation [41].

### 2.4. Dimeric-Helicase and Monomeric-Helicase Models for SF1 and SF2 Helicases Other than E. coli SF1 UvrD Helicase

This paper focuses on *E. coli* SF1 UvrD helicase and describes studies that proposed dimeric-helicase and monomeric-helicase models for UvrD helicase. For the sake of comparison and better understanding, this section deals with *B. subtilis* SF1 PcrA and *E. coli* SF2 RecQ helicases, for which both dimeric-helicase and monomeric-helicase models have been proposed, and provides a brief overview of the studies that proposed the models.

#### 2.4.1. PcrA

For SF1 PcrA helicase, a monomeric helicase model was proposed based on the obtained crystal structures of monomeric PcrA-DNA complexes, with the result that only the fraction of PcrA monomer was detected by size-exclusion chromatography [9]. On the other hand, Yang et al. indicated that PcrA functions as a dimeric form through single- and multiple-turnover DNA-unwinding experiments [25]. They showed that DNA unwinding by PcrA was promoted by increasing the PcrA concentration and that the Hill coefficient in unwinding and ATPase reactions was about 2. Niedziela-Majka et al. showed through single-turnover DNA-unwinding experiments that PcrA monomers exhibited excellent single-stranded DNA (ssDNA) translocase activity but no detectable helicase activity [42]. In contrast, Chistry et al. suggested by single-molecule measurements that PcrA monomer can unwind double-stranded DNA (dsDNA) of more than 1 kbase pair (bp) length in the presence of its partner protein RepD [33].

#### 2.4.2. RecQ

For SF2 RecQ helicase, Xu et al. indicated that the helicase unwinds DNA in monomeric form [37]. They reached this conclusion from the following results: (i) immunoprecipitation experiments detected no interaction between RecQ monomers; (ii) size exclusion chromatography and analytical-sedimentation–equilibrium-ultracentrifugation experiments suggested that RecQ helicase exists as a monomer in solution; and (iii) stopped-flow experiments detected no increase in the DNA unwinding rate with the increase in RecQ helicase concentration. Li et al. performed fluorescence cross-correlation spectroscopy (FCCS) and reported that a RecQ monomer was capable of unwinding short DNA substrates, which supports the monomeric model [39]. They also reported that multiple RecQ monomers simultaneously could bind to long DNA substrates and suggested that these RecQ monomers unwound the DNA quite efficiently using “functional cooperativity”. They then concluded that the length of DNA substrates, the number and length of the 3′ ssDNA tail, and the temperature affect the functional cooperativity. This DNA length effect coincided with the results using single-molecule fluorescence imaging reported by Rad et al. [26]. They used λ DNA (48.5 kbp) and revealed that the unwinding rate of RecQ increased with the increase in RecQ concentration, which was different from the results reported by Xu et al. using short DNA substrates and suggested that a RecQ dimer was responsible for the initiation of DNA unwinding.

## 3. C-Terminal Amino Acids

The seven helicase motifs of SF1 and SF2 helicases, which are essential to ATP hydrolysis and nucleic acid binding and unwinding, are commonly placed in the middle of the amino acid sequences. In contrast, the N-terminal and C-terminal amino acids outside of helicase motifs exhibit large variations in their sequence and length, though these terminal regions are in charge of various protein functions.

Past studies showed that the C-terminus is crucial to nucleic acid binding and unwinding and dimer formation. These include the bacteriophage P4 gpα helicase-primase, the yeast Rad25 helicase [43,44], and Werner’s syndrome protein [45,46]. The roles of the C-terminus have been well studied for SF2 DEAD-box RNA helicases that are engaged in RNA metabolism [47]. For example, the C-terminal region of CshA and Hera served dual functions of dimerization and RNA binding [30,31]. Table 2 lists the reported roles of C-terminal amino acids on SF2 DEAD/DEAH-box RNA helicases.

## 4. C-Terminus Truncated UvrD

*E. coli* SF1 UvrD helicase (720 amino acids) assumes a pivotal role in both nucleotide-excision repair and methyl-directed mismatch repair [52]. Like other SF1 helicases, UvrD consists of four structural domains (1A, 1B, 2A, and 2B) [6] and has an unstructured C-terminal region (645–720 amino acids) [6,53]. The UvrD protein is a 3′ to 5′ helicase and unwinds dsDNA from the 3′ end ssDNA tail using energy derived from ATP hydrolysis. Past biochemical studies have indicated that this helicase exhibits optimal DNA unwinding activity in its oligomeric form [16].

However, X-ray crystallographic structures of monomeric UvrD [6] and results from genetic and biochemical assays provided the opposite monomeric-helicase model [32,54]. In these studies, UvrDΔ40C was used. Mechanic et al. found, via genetic-complementation assays using a strain lacking the *uvrD* gene, that UvrDΔ40C was competent enough to fulfill methyl-directed mismatch repair and nucleotide-excision repair. They also examined self-interaction between UvrDΔ40C molecules with a yeast two-hybrid system and reported that UvrDΔ40C was unable to dimerize. In addition, they found that UvrDΔ40C retained comparable ssDNA-binding, ssDNA-stimulated ATPase, and DNA-unwinding activities, compared to wild-type UvrD. They reported the following results: (i) the affinity of ATP to UvrDΔ40C (*K_m_* = 62 μM) was almost as same as that to wild-type UvrD (*K_m_* = 50 μM); (ii) the turnover rate for ssDNA-stimulated ATP hydrolysis for UvrDΔ40C (*k*_cat_ = 147 s^−1^) was almost the same as that for wild-type UvrD (*k*_cat_ = 157 s^−1^); and (iii) UvrDΔ40C unwound 92-bp and 234-bp partial duplex DNA substrates as efficiently as wild-type UvrD. They also indicated that UvrDΔ40C could not dimerize in their results (obtained by size-exclusion chromatography and analytical-sedimentation–equilibrium-ultracentrifugation experiments). The elution pattern of UvrDΔ40C in size-exclusion chromatography showed only a single peak that corresponded to the UvrDΔ40C monomer. Molecular mass that corresponded to dimeric UvrDΔ40C was not detected in analytical-sedimentation–equilibrium-ultracentrifugation experiments even though the UvrDΔ40C concentration was increased. Therefore, they proposed the monomeric model. Table 3 lists the reported effects of deleting C-terminal amino acids from UvrD in its oligomerization state.

UvrD mutants lacking longer C-terminal amino acids (UvrDΔ73C, UvrDΔ102C, or UvrDΔ107C) were also studied [32,53,54]. UvrDΔ102C and UvrDΔ107C were incompetent and could not perform DNA repair. UvrDΔ73C exhibited a slightly reduced ssDNA binding affinity, whereas UvrDΔ102C displayed a considerably reduced affinity. The ssDNA-binding affinity of the C-terminal deletion mutants corresponded with their ATPase and DNA-unwinding abilities. UvrDΔ73C kept its abilities, but UvrDΔ102C did not. They reported the following results: (i) the ssDNA binding affinity of UvrDΔ73C (*K_m_* = 2.5 μM) was more than four times lower than that of wild-type UvrD (*K_m_* = 0.54 μM); (ii) the turnover rate for the ssDNA-stimulated ATP hydrolysis for UvrDΔ73C (*k*_cat_ ~ 500 s^−1^) was almost the same as that for wild-type UvrD (*k*_cat_ ~ 400 s^−1^). The UvrDΔ102C mutant was void of both the unstructured C-terminus (645–720 amino acid) and some of the 2A domain [6]. Thus, the unstructured C-terminal region is supposed to be the necessity for DNA unwinding, and the conserved 2A domain should be more important for activity. Then, it is probable that the length of the unstructured C-terminal region affects the DNA-unwinding ability. Maluf et al. described the potential for UvrDΔ40C to dimerize, as they illustrated that UvrDΔ73C was able to dimerize [41]. They showed that UvrDΔ73C did dimerize by analytical-sedimentation–equilibrium-ultracentrifugation experiments, but the dimerization equilibrium constant was 25 times smaller than that for wild-type UvrD. Table 4 summarizes the reported effects of deleting C-terminal amino acids from UvrD on its functions.

## 5. Single-Molecule Direct Visualization of UvrDΔ40C

In comparison with conventional biochemical and genetic studies that provide data on ensemble averages of multi-molecules, single-molecule fluorescence imaging can assess the real-time behavior of non-averaged individual biomolecules, allowing us to elucidate their detailed dynamical features. [55].

Therefore, single-molecule direct visualization of UvrDΔ40C was performed to address how the C-terminal amino acids affect the number of helicases bound to DNA, oligomerization, and DNA-unwinding activity.

### 5.1. Observation of Multiple UvrDΔ40C Molecules that Bound to DNA in the Absence of ATP

**Figure 3:**
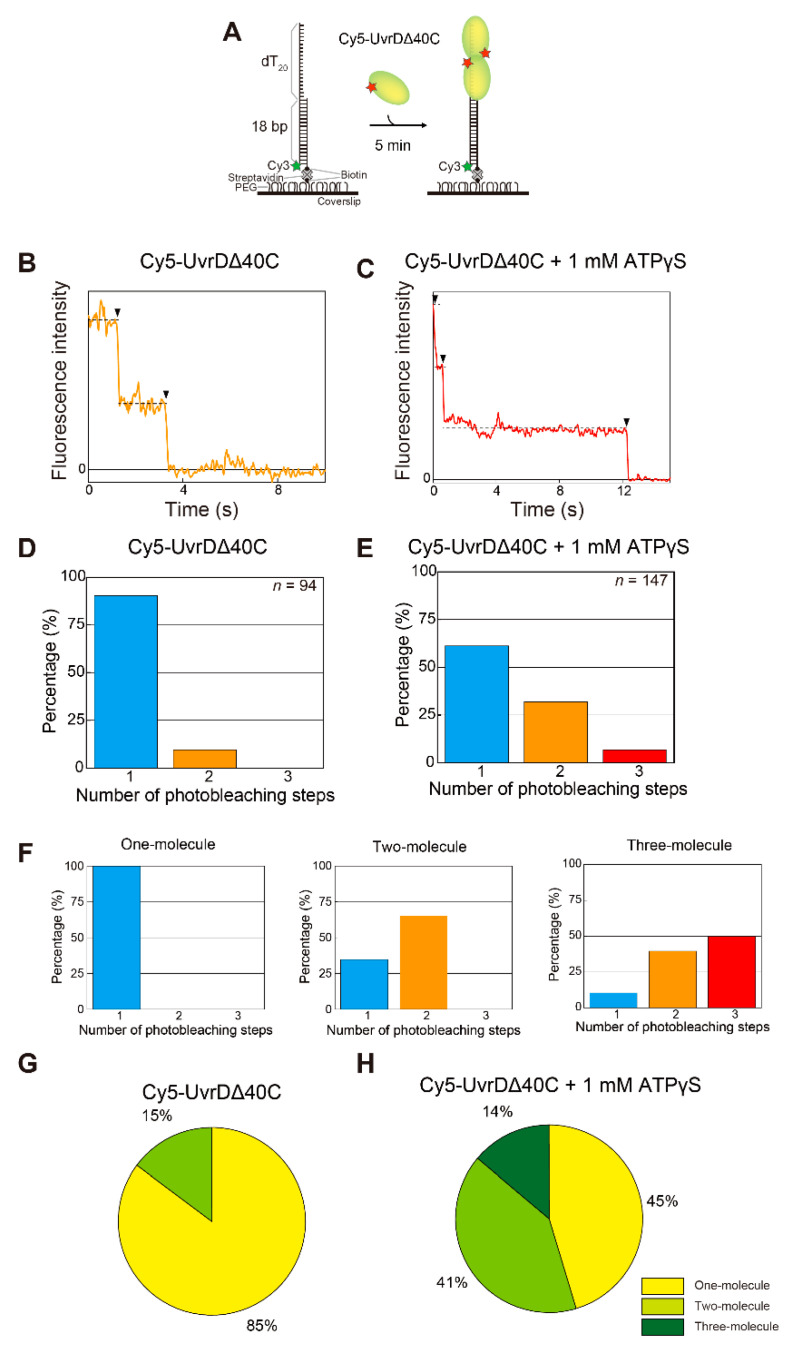
Single-molecule visualization of Cy5-UvrDΔ40C bound to a DNA substrate in the absence of adenosine triphosphate (ATP): (**A**) schematic drawing of the assay; (**B**,**C**) the photobleaching processes (indicated by arrowheads) of the fluorescent Cy5-UvrDΔ40C spots for two steps for UvrDΔ40C alone (**B**) and three steps for UvrDΔ40C and 1 mM adenosine 5′-(γ-thio)triphosphate (ATPγS), respectively (**C**); (**D**,**E**) experimentally obtained distributions of the number of photobleaching steps. The total number of analyzed fluorescent spots is indicated for each condition; (**F**) theoretical distributions of the number of photobleaching steps for the one-molecule, two-molecule, and three-molecule models; (**G**,**H**) pie charts showing the percentages of each theoretical model, as predicted by the linear combination for (**G**) Cy5-UvrDΔ40C alone and for (**H**) Cy5-UvrDΔ40C and 1 mM ATPγS. Reproduced with permission from [10]. Copyright 2020, Biophysical Society.

To investigate whether or not UvrDΔ40C binds to DNA in only a monomeric form, single-molecule quantification of UvrDΔ40C molecules that bind to DNA was performed under the condition of UvrDΔ40C molecules alone or in the presence of both UvrDΔ40C molecules and adenosine 5′-(γ-thio)triphosphate (ATPγS), a non-hydrolyzable ATP analog. The assay utilized an 18-bp dsDNA with a 20-nucleotide (nt) 3′ ssDNA tail [16,41,56,57,58]. The DNA was immobilized on the glass surface through streptavidin–biotin interactions and visualized by the fluorescence of Cy3 attached to one of the oligonucleotides of the DNA [18] (Figure 3A). Then, a Cy5-labeled Cys-Ala mutant (Cy5-UvrDΔ40C) was infused, and the number of Cy5 photobleaching steps at each Cy3-DNA site was counted using a dual-view apparatus that enabled simultaneous two-color single-molecule imaging. The UvrDΔ40C mutant only contained single inherent Cys (Cys^52^) and thus was labeled with a single Cy5 molecule, with high specificity and a high labeling ratio of 79%. Buffer U (6 mM NaCl, 2.5 mM MgCl_2_, 10% (*v*/*v*) glycerol, and 25 mM Tris-HCl (pH 7.5)) was used in all of the experiments in this section [18,58] unless otherwise mentioned. Figure 3B,C shows a two-step photobleaching event observed in the presence of Cy5-UvrDΔ40C alone in solution and a three-step photobleaching event observed in the presence of both Cy5-UvrDΔ40C and 1 mM ATPγS in solution, respectively. The experimentally obtained distributions of the number of photobleaching steps are shown in Figure 3D,E. The fraction of the two photobleaching steps in Figure 3D and the fraction of the three photobleaching steps in Figure 3E demonstrate that at least two or three UvrDΔ40C molecules can bind DNA under the corresponding solution conditions. Moreover, these results suggest that the presence of ATPγS made more UvrDΔ40C molecules bind to DNA, which was observed for wild-type UvrD [18].

The predicted distributions of the number of photobleaching steps are shown in Figure 3F. The distributions were calculated based on the labeling ratio of Cy5-UvrDΔ40C (79%) for the one-molecule, two-molecule, or three-molecule models of UvrDΔ40C binding to DNA. Note that non-labeled UvrDΔ40C (21%) was invisible by single-molecule fluorescence imaging. Thus, the percentages of the models contained in the experimentally obtained distributions were obtained by fitting with a linear combination of the theoretical models. Figure 3G,H shows the percentages of the models, indicating that multiple UvrDΔ40C molecules could bind to DNA.

### 5.2. Observation of Multiple UvrDΔ40C Molecules’ Association to DNA, which Synchronized with the Unwinding Activity in the Presence of ATP

**Figure 4:**
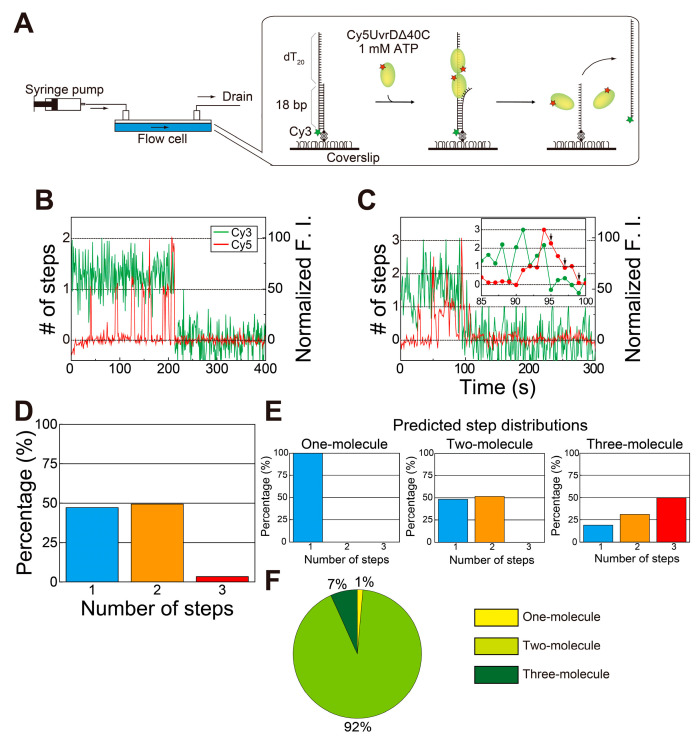
Simultaneous single-molecule visualization of the association/dissociation of Cy5-UvrDΔ40C with/from a double-stranded DNA (dsDNA) substrate and unwinding of DNA in the presence of ATP: (**A**) Schematic drawing of the assay; (**B**,**C**) typical time traces of the Cy3- and Cy5-fluorescence intensities (F.I.), where the Cy5-fluorescence intensity increased in a two-step manner (**B**) and a three-step manner (indicated with arrows) (**C**) just before DNA unwinding, resulting in the Cy3 fluorescence disappearance (**D**), respectively; (**D**) experimentally obtained distribution of the number of step changes in the Cy5 fluorescence; (**E**) theoretical distributions of the number of Cy5 fluorescence steps relevant to the completion of DNA unwinding processes; (**F**) pie chart showing the percentages of each theoretical model, as predicted by linear combination for (**E**). Reproduced with permission from [10]. Copyright 2020, Biophysical Society.

Next, the number of UvrDΔ40C molecules bound to the DNA and DNA unwinding by the molecules in the presence of ATP was simultaneously monitored by single-molecule visualization (Figure 4A). Completion of DNA unwinding was detectable through the disappearance of Cy3 fluorescence.

As observed for wild-type UvrD [18], two- or three-step Cy5 fluorescence changes were observed immediately before finishing unwinding DNA (Figure 4B,C). These observations provide insights into the DNA-unwinding mechanism of UvrDΔ40C. UvrDΔ40C unwinds DNA in the same fashion as wild-type UvrD [18]: DNA unwinding is completed by multiple UvrDΔ40C molecules. In fact, the ratios of the step numbers for the Cy5 fluorescence changes (Figure 4D) are very similar to the two-molecule model (Figure 4E). The dwell time for the second UvrDΔ40C association, as well as the estimated number of Cy5 molecules bound per UvrDΔ40C molecule, were used to work out the ratios of the models. Moreover, the distribution (Figure 4D) was fitted by a linear combination of the theoretical models. The fitting shows that the percentage of the one-molecule model is only 1% (Figure 4F), demonstrating that two UvrDΔ40C molecules were engaged in complete DNA unwinding in most cases and that three UvrD Δ40C molecules also took part in the process in some cases.

**Figure 5:**
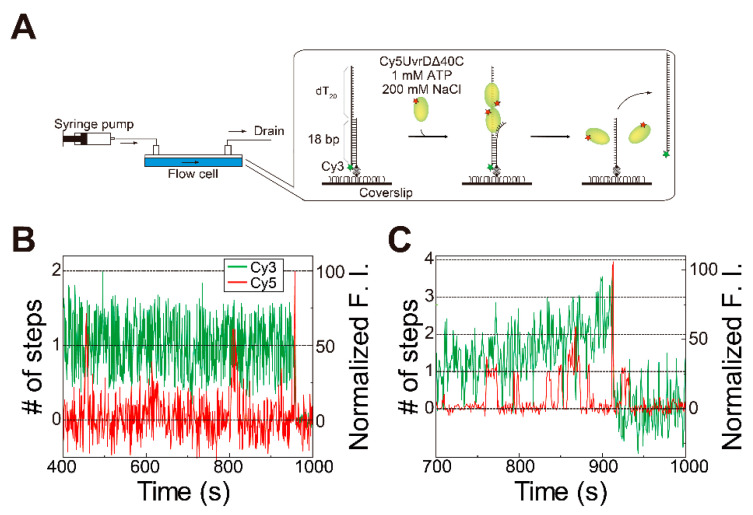
Simultaneous single-molecule visualization of the association/dissociation of Cy5-UvrDΔ40C with/from a dsDNA substrate and unwinding of the DNA in the presence of ATP and 200 mM NaCl in solution: (**A**) schematic drawing of the assay; (**B**,**C**) time traces of the Cy3- and Cy5-fluorescence intensities (F.I.), where a Cy5-fluorescence intensity increase corresponding to two Cy5-UvrDΔ40C molecules was observed just before DNA-unwinding completion (Cy5-UvrDΔ40C concentration = 2 nM) (**B**) and where a Cy5-fluorescence intensity increase corresponding to more than two Cy5-UvrDΔ40C molecules was observed just before DNA-unwinding completion (Cy5-UvrDΔ40C concentration = 10 nM) (**C**), respectively. Reproduced with permission from [10]. Copyright 2020, Biophysical Society.

Cy5 fluorescence increases corresponding to two or more Cy5-UvrDΔ40C molecules were also observed under physiologically relevant, high-salt buffer conditions (200 mM NaCl) (Figure 5), suggesting that multiple UvrDΔ40C molecules were actually involved in DNA unwinding in vivo, though dimer formation is difficult under these high-salt conditions.

### 5.3. Multiple UvrDΔ40C Molecules that Bound to the DNA Are Likely to Form an Oligomer

Multiple UvrDΔ40C molecules that unwound DNA were likely to directly self-interact on ssDNA and oligomerize, which was initially proposed from a non-linear sigmoidal dependence of DNA unwinding efficiency on the ratio of wild-type UvrD concentration to DNA concentration [16].

**Figure 6:**
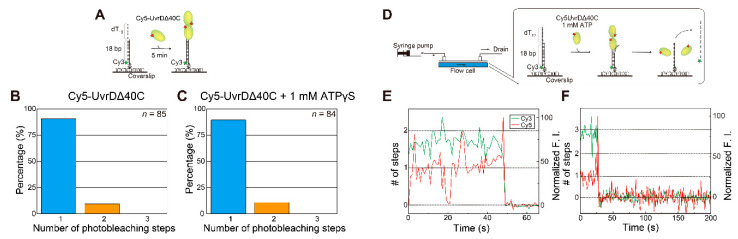
Single-molecule visualization of Cy5-UvrDΔ40C bound to a 18-base pair (bp) dsDNA substrate with a 12-nt 3′ single-stranded DNA (ssDNA) tail in the absence or the presence of ATP: (**A**) schematic drawing of the assay for the absence of nucleotides or the presence of 1 mM ATPγS; (**B**,**C**) experimentally obtained distributions of the number of photobleaching steps for the Cy5-UvrDΔ40C that bound to an 18-bp DNA with a 12-nt 3′ ssDNA tail in solutions containing 2 nM Cy5-UvrDΔ40C alone (**B**) and 2 nM Cy5-UvrDΔ40C and 1 mM ATPγS (**C**), respectively. The total number of analyzed fluorescent spots is indicated for each condition; (**D**) schematic drawing of the assay for the presence of 1 mM ATP; (**E**,**F**) simultaneous single-molecule visualization of the association/dissociation of Cy5-UvrDΔ40C with/from the dsDNA substrate and unwinding of the DNA in a solution containing 10 nM Cy5-UvrDΔ40C and 1 mM ATP; (**E**) time traces of the Cy3- and Cy5-fluorescence intensities (F.I.), where the Cy5-fluorescence intensity increased in a two-step manner just before DNA unwinding resulting in the Cy3 fluorescence disappearance; (**F**) time traces of the Cy3- and Cy5-fluorescence intensities, where the maximum Cy5-fluorescence intensity was supposed to come from three Cy5-UvrDΔ40C molecules just before the DNA unwinding process. Reproduced with permission from [10]. Copyright 2020, Biophysical Society.

To test this self-interaction hypothesis, single-molecule photobleaching step analysis (Figure 3) was performed using a dsDNA substrate with a shorter 3′ ssDNA tail (18-bp DNA with a 12-nt 3′ ssDNA tail) in the absence of ATP (Figure 6A). The number of UvrDΔ40C molecules that bound to this DNA substrate was supposed to decrease [18,56] since a lower limit of the estimated wild-type UvrD site size on poly(dT) was reported to be 10 ± 2 nt [59] and wild-type UvrD molecules were reportedly hard to bind to blunt dsDNA [18]. However, the analysis revealed that two UvrDΔ40C molecules were bound even to the DNA (Figure 6B,C). Moreover, the single-molecule DNA-unwinding assay (Figure 4) using the DNA substrate in the presence of ATP demonstrated (Figure 6D) that multiple UvrDΔ40C molecules still participate in the DNA-unwinding process (Figure 6E,F). These results strongly support the self-interaction or oligomerization of UvrDΔ40C molecules on DNA.

The 3′ ssDNA length (12 nt) was reported to be the minimum length to complete unwinding dsDNA as long as 18 bp [16], which implies that oligomerization of the multiple UvrDΔ40C molecules along ssDNA is crucial to unwinding DNA. Nguyen et al. showed that the 2B domain of the UvrD that first bound to DNA was altered to a more closed conformation by the binding of a second UvrD to the first bound UvrD (dimerization), activating DNA-unwinding activity [21]. These findings also support that multiple UvrDΔ40C molecules that were involved in DNA unwinding made some physical contact.

The opposing “independent monomer” model has been proposed for some non-hexameric helicases such as RecQ [26] and Dda [36]. This model was built based on the notion that multiple helicases participate in DNA unwinding, but they unwind DNA without self-interaction or oligomerization. Note that this model does not exclude some interaction between helicases. Although UvrD might unwind DNA in this manner, this model was not applicable to UvrD [16].

### 5.4. Transient Two UvrD Bound State Just before Completion of the DNA-Unwinding Process

**Figure 7:**
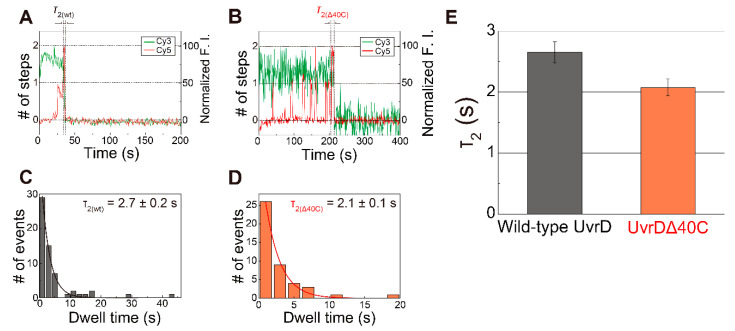
The dwell time (*τ*_2_) between the appearance of the second step and completion of the DNA-unwinding process: (**A**,**B**) typical time traces of the Cy3- and Cy5-fluorescence intensities (F.I.) for Cy5- labeled wild-type UvrD (**A**) and Cy5- labeled UvrDΔ40C (**B**) (Figure 4B), respectively, where each dwell time (*τ*_2(wt)_ and *τ*_2(Δ40C)_) is indicated; (**C**,**D**) *τ*_2_ distributions for Cy5- labeled wild-type UvrD (**C**) and Cy5- UvrDΔ40C (**D**), respectively, the mean dwell time, which was obtained by single exponential fittings were 2.7 ± 0.2 s for Cy5- labeled wild-type UvrD and 2.1 ± 0.1 s for Cy5-UvrDΔ40C; (**E**) comparison of the obtained *τ*_2_. The error bars represent the standard errors. Reproduced with permission from [10,18]. Copyright 2013, 2020, Biophysical Society.

The dwell time between the appearance of the second step of the Cy5 fluorescence increase and the completion of the DNA-unwinding process, which is defined as τ_2_, is indicated in Figure 7A,B for Cy5- labeled wild-type UvrD and Cy5-UvrDΔ40C (as same as Figure 4B), respectively. Figure 7C,D shows their τ_2_ distributions.

The two UvrDΔ40C molecules that bound to the DNA are likely to form an oligomer. Then, three kinetic steps are supposed to take place during the mean dwell time of two UvrDΔ40C-bound states (τ_2(Δ40C)_ = 2.1 ± 0.1 s). These are: (i) late-coming UvrDΔ40C molecule(s) translocated along ssDNA and forming an oligomer with earlier bound UvrDΔ40C molecule(s); (ii) the oligomer isomerized to become a productive oligomer that was prepared for unwinding DNA; and (iii) the isomerized oligomer unwinding DNA. Since UvrDΔ40C has similar helicase activity to wild-type UvrD [32,54], UvrDΔ40C might translocate along ssDNA at the same speed as wild-type UvrD (translocation rate along dT of ~190 nt s^−1^ [60] and a processivity of 769 ± 1 nt [61]) and can encounter pre-bound UvrDΔ40C monomer(s) in less than 0.1 s. Then, since the unwinding rate and the processivity of wild-type UvrD are reported to be 68 ± 9 bp/s [16] and 40–50 bp [62], respectively, the productive UvrDΔ40C oligomer must complete unwinding the 18-bp dsDNA substrate in <0.5 s without dissociating from the DNA. To sum up, unwinding of the 18-bp dsDNA substrate would be completed in less than 0.5 s after the late-coming UvrDΔ40C monomer bound to the DNA.

Interestingly, UvrDΔ40C has a mean τ_2_ shorter than wild-type UvrD [18] (Figure 7E). This result suggests that UvrDΔ40C molecules isomerize faster than wild-type UvrD molecules, which is consistent with the result by Mechanic et al., in which UvrDΔ40C molecules had a slightly higher DNA unwinding rate than wild-type UvrD molecules [32]. Therefore, the C-terminal 40-amino acid deletion should alter the isomerization process in some way.

### 5.5. Association/Dissociation Rates for the UvrDΔ40C–DNA Interaction

**Figure 8:**
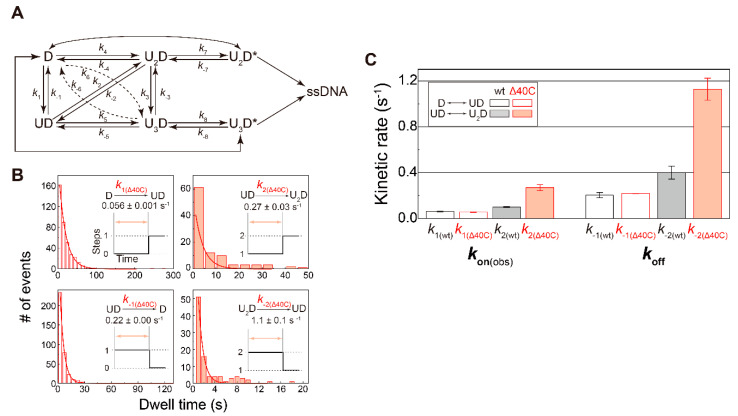
Association- and dissociation-rate constants: (**A**) kinetic scheme of the UvrD–DNA interaction. U and D represent UvrD and DNA, respectively; (**B**) dwell-time distributions of the indicated states. Association- and dissociation-rate constants were obtained by single exponential fit of each distribution. *k*_1_ and *k*_2_ are association rate constants under 2 nM UvrDΔ40C concentration; (**C**) comparison of the obtained rate constants between UvrDΔ40C and wild-type UvrD. *K*_on(obs)_ are the observed association rate constants under 2 nM UvrDΔ40C concentration. The error bars represent standard errors. Reproduced with permission from [10]. Copyright 2020, Biophysical Society.

The observed single-molecule UvrDΔ40C association/dissociation events (Figure 4B,C) enabled the determination of the kinetic features of the UvrDΔ40C–DNA interaction. The traces with stepwise Cy5 fluorescence increases or decreases were considered to be UvrDΔ40C associations or dissociations. The kinetic scheme of UvrD–DNA interactions is shown in Figure 8A, which was proposed in the author’s past single-molecule direct visualization for wild-type UvrD [18].

The dwell-time distributions for some kinetic steps are shown in Figure 8B. Dwell time for the dissociation events immediately before completion of the DNA-unwinding process was excluded from the distributions. Each single exponential fitting provided the corresponding rate constants. Note that the more UvrDΔ40C molecules were involved in the association/dissociation processes, the higher rate constants were estimated, which was already observed for wild-type UvrD (Figure 8C).

For the first bound UvrD, UvrDΔ40C exhibited association and dissociation rates comparable to wild-type UvrD. In contrast, for the second bound UvrD, UvrDΔ40C had higher rates (approximately 2.5-fold higher than wild-type UvrD). These results represented that the lifetime of a UvrDΔ40C oligomer was shorter than that of wild-type UvrD.

## 6. Summary

This paper has given an overview of past and recent studies that proposed helicase mechanisms and investigated the effect of C-terminal amino acid truncation on helicase and dimerization activities, primarily on the non-hexameric *E. coli* SF1 UvrD helicase. Among non-hexameric SF1 and SF2 helicases, the roles of the C-terminus were well studied for SF2 DEAD-box helicases. These studies showed that the C-terminus is a key region of nucleic acid binding and dimerization activities that are directly linked to the DNA unwinding function. To illustrate how the C-terminus affects the functions of SF1 helicases, I have introduced my latest single-molecule direct visualization study on UvrDΔ40C, which was used for studies proposing the monomer helicase model, and shown how the truncation of C-terminal amino acids outside the conserved helicase domains affects the helicase–DNA interaction and dimerization activities. Contrary to the proposed model, it has been shown that multiple UvrDΔ40C molecules jointly participated in DNA unwinding, presumably by forming an oligomer. Thus, single-molecule observations can help us increase our understanding of protein–nucleic acid interactions. It can also identify transient and minor populations that are usually overlooked by conventional ensemble averaging-based measurements and can determine kinetic rate constants.

Other single-molecule observations by single-molecule DNA-manipulation [17] and by direct visualization of single UvrD molecules [19] from other groups support the oligomer model. The possible self-interaction or oligomer formation of UvrD discussed in this review was also indicated by these single-molecule studies. In addition, data from a recent single-molecule fluorescence resonance energy transfer (FRET) study showed that the 2B domain of the UvrD that first bound to DNA was altered to a more closed conformation by the binding of a second UvrD to the first bound UvrD (dimerization), activating DNA-unwinding activity [21]. The conformational change in the 2B domain, another significant feature of SF1 helicases, was visualized by single-molecule force-fluorescence microscopy, in which single-molecule visualization of wild-type UvrD molecules and DNA-unwinding activity monitoring with optical tweezers were simultaneously feasible [20].

The single-molecule UvrDΔ40C visualization study introduced in this review, which can be applied to other helicases, addressed how C-terminal amino acids affect the number of helicases bound to DNA, oligomerization, and unwinding activity. Single-molecule microscopy such as single-molecule visualization and single-molecule DNA manipulation will serve as a means to explore how the DNA-binding and DNA-unwinding activities of helicases are affected by their C-terminus and partner proteins.

## Figures and Tables

**Table 1:** Cellular functions of some SF1 and SF2 helicases and the proposed models of their functional units.

SF	Helicase	Organism	Cellular Functions	Proposed Models for Functional Unit	References
SF1	UvrD	*E. coli*	DNA repair	Monomer/Dimer	[6,10,16,17,18,19,20,21,32]
SF1	Rep	*E. coli*	DNA replication	Dimer	[8,22,23]
SF1	TraI	*E. coli*	DNA transfer during conjugation	Monomer/Dimer	[24,34]
SF1	PcrA	*B. subtilis*	DNA repair	Monomer/Dimer	[9,25,33]
			Rolling replication of plasmids		
SF1	Dda	Phage T4	DNA replication initiation	Monomer	[35,36]
			DNA recombination		
SF2	RecQ	*E. coli*	DNA recombination	Monomer/Dimer	[26,37,38,39]
SF2	CsdA	*E. coli*	Ribosome biogenesis	Dimer	[28]
SF2	RhlB	*E. coli*	RNA metabolism	Dimer	[29]
SF2	NS3	Hepatitis C virus	Viral DNA replication	Monomer/Dimer	[27,40]
SF2	CshA	*G. stearothermophilus*	RNA metabolism	Dimer	[30]
SF2	Hera	*T. thermophilus*	RNA metabolism	Dimer	[31]

**Table 2:** Reported roles of C-terminal amino acids on SF2 Asp-Glu-Ala-Asp (DEAD)/Asp-Glu-Ala-His (DEAH) box RNA helicases.

Subfamily	Helicase	Organism	Roles of C-Terminal Amino Acids	References
DEAD-box	CsdA	*E. coli*	RNA binding	[28]
DEAD-box	CshA	*G. stearothermophilus*	RNA binding, RNA-dependent ATP hydrolysis, and interaction with degradosome	[30,48]
DEAD-box	Hera	*T. thermophilus*	RNA binding and dimerization	[31]
DEAD-box	Mss116p	*S. cerevisae*	RNA-dependent ATPases	[49]
DEAD-box	YxiN	*B. subtilis*	RNA binding	[50]
DEAD-box	p68	*Homo sapiens*	RNA binding	[51]

**Table 3:** Effects of deleting C-terminal amino acids from UvrD on its oligomerization state.

UvrD	Size-Exclusion Chromatography	Sedimentation Equilibrium Experiments	Velocity Equilibrium Experiments
UvrDΔ40C	Monomer [32]	Monomer [32]	Monomer [32]
UvrDΔ73C	N.D.	Dimer [41]	N.D.

N.D.: not determined.

**Table 4:** Effects of deleting C-terminal amino acids from UvrD on its functions.

UvrD	DNA Repair	DNA-Stimulated ATPase	DNA Binding	DNA Unwinding
UvrDΔ40C	+ ^1^ [32,54]	+ [32,54]	+ [54]	+ [32,54]
UvrDΔ73C	+ ^2^ [53]	+ [53]	+ [53]	+ [53]
UvrDΔ102C	− ^1^ [54]	− [54]	− [54]	− [54]
UvrDΔ107C	− ^1^ [54]	N.D.	N.D.	N.D.

^1^ Experiments were performed in vivo. ^2^ Experiments were performed in vitro.+: activity was detected. −: activity was not detected. N.D.: not determined.

## Data Availability

The data that support the findings of this study are available from the corresponding author upon reasonable request.

## Abbreviations

ATP	adenosine triphosphate
ATPγS	adenosine 5′-(γ-thio)triphosphate
*B. stearothermophilus*	*Bacillus stearothermophilus*
bp	base pair
dsDNA	double-stranded DNA
*E. coli*	*Escherichia coli*
F.I.	fluorescence intensities
FCCS	fluorescence cross-correlation spectroscopy
FRET	fluorescence resonance energy transfer
*G. stearothermophilus*	*Geobacillus stearothermophilus*
N.D.	not determined
nt	nucleotide
SF	superfamily
ssDNA	single-stranded DNA
*T. thermophilus*	*Thermus thermophilus*
UvrDΔ102C	UvrD mutant lacking the C-terminal 102 amino acids
UvrDΔ107C	UvrD mutant lacking the C-terminal 107 amino acids
UvrDΔ40C	UvrD mutant lacking the C-terminal 40 amino acids
UvrDΔ73C	UvrD mutant lacking the C-terminal 73 amino acids
